# Supplementary material for: Inhibition of the Notch signaling pathway promotes AQP2 plasma membrane accumulation in renal epithelial cells by depolymerizing actin and reducing endocytosis
Source: bioRxiv. 2026 Aug 12:2026.08.11.744289. Preprint. [Version 1] doi: 10.64898/2026.08.11.744289 (PMC13640677; doi:10.64898/2026.08.11.744289)
Supplement: Supplementary file 1 [file media-1.pdf]

Supplemental table: List of Antibodies

Antigen	Manufacturer	Catalog	References	Dilution (WB)
RhoA	Cell Signaling	2117T	PMID:37730597	1:1000
GAPDH	Applied Biosystems	4300	PMID: 32434945	1:10000
Cleaved Notch 1	Cell Signaling	4147	PMID: 40190020	1:1000
P256 AQP2	Affinity	3673	PMID: 15509592	1:1000
P269 AQP2	Cell Signaling	3674	PMID: 15509592	1:1000
P261 AQP2	Rockland antibodies	612-401 D08	PMID: 7508187	1:1000
AQP2	Santa Cruz	9882	PMID: 22218592	1:1000

Supplemental Figure 1:

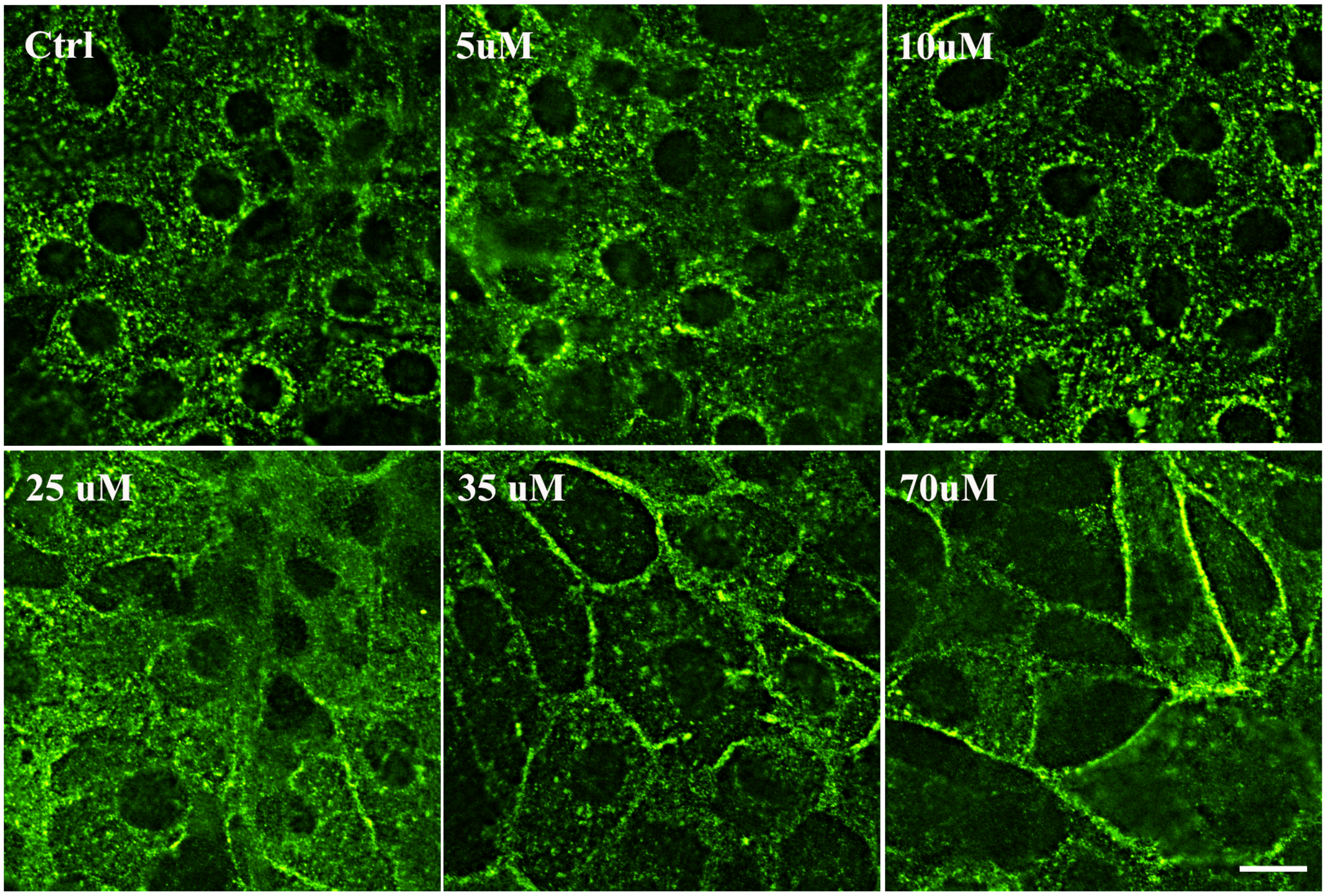

Supplementary Figure 1. DAPT induces membrane accumulation of AQP2 in a dose-dependent manner in LLC-AQP2 cells. Immunofluorescence staining of AQP2 with anti-c-myc antibody in LLCAQP2 cells treated with DAPT at various concentrations (5, 10, 25, 35 and 70) μM for 30 min. Membrane accumulation of AQP2 was detectable in cells treated with 25 μM DAPT and peaked at 35 μM DAPT. Scale bar = 10 μm.
